# Multiple system atrophy: integrating clinical phenotypes and multimodal biomarkers for early diagnosis

**DOI:** 10.3389/fnagi.2026.1801658

**Published:** 2026-05-29

**Authors:** Ida Wilkens, Franziska Hopfner

**Affiliations:** Department of Neurology, LMU University Hospital, Ludwig-Maximilians-Universität München, Munich, Germany

**Keywords:** α-synuclein, cerebellar ataxia, imaging, MSA, multiple system atrophy, pure autonomic failure (PAF), seed amplification assay, synucleinopathies

## Abstract

Multiple system atrophy is a rare and rapidly progressive synucleinopathy characterized by parkinsonism, cerebellar ataxia, and prominent autonomic dysfunction. Neuropathological findings include misfolded α-synuclein accumulation in oligodendrocytes and neuronal loss leading to multisystem neurodegeneration. As a definite diagnosis still requires postmortem confirmation, there is a critical need for reliable *in vivo* diagnostic and prognostic biomarkers, particularly for early and prodromal disease stages in the context of upcoming disease-modifying therapies. This review provides a comprehensive overview of current concepts in the diagnosis of MSA with a particular focus on established and emerging biomarkers. It summarizes the clinical diagnostic framework and functional testing that support differential diagnosis in routine practice. Furthermore, it discusses the role of imaging biomarkers, ranging from conventional MRI to advanced multimodal MRI-techniques and molecular imaging approaches such as PET markers of neuroinflammation and α-synuclein pathology. In addition, it reviews the current state of fluid biomarkers such as seed amplification assays and neurofilament light chain and addresses biomarker-based strategies for disease monitoring and patient stratification in clinical trials. Overall, this review aims to integrate literature of clinical, imaging, and fluid biomarkers into a multimodal framework to improve early diagnosis, support biological classification, and facilitate therapeutic development in MSA.

## Introduction

Multiple System atrophy (MSA) is a rare, neurodegenerative disorder leading to a variable spectrum of the main symptom complexes parkinsonism, cerebellar ataxia and autonomic dysfunction (Halliday et al., 2011). The disease usually manifests in the 6th life decade and is marked by a rapid progression and an average life expectancy of 5–9 years after diagnosis (Low et al., 2015). As a synucleinopathy, MSA leads to the accumulation of misfolded α-synuclein, which drives progressive neurodegeneration within the striatonigral, olivopontocerebellar, and autonomic nervous systems (Wiseman et al., 2025a; Jellinger and Lantos, 2010). Histologic hallmarks include glial cytoplasmic inclusions (GCIs), consisting of aggregated and misfolded α-synuclein (Trojanowski and Revesz, 2007; Papp and Lantos, 1994). The density of GCIs correlates with the degree of neurodegeneration (Trojanowski and Revesz, 2007). In contrast to Parkinson's disease (PD) and dementia with Lewy bodies (DLB), glial pathology is central in MSA, and the disorder is therefore classified as an oligodendroglial synucleinopathy (Trojanowski and Revesz, 2007).

At present, there are no reliable biomarkers that allow a definite diagnosis in living patients. The final diagnosis is based on histopathological examination of postmortem brain tissue. Hence, current research focuses on biomarkers that may help to diagnose MSA, especially in the early stages of the disease, potentially even at the premotor stage. This becomes even more urgent with the emergence of potential therapeutic studies that may modify the course of MSA.

## Pathogenesis

The pathogenesis of MSA is not clear yet. To date, no environmental factors influencing the risk of developing MSA have been identified. Consequently, MSA is considered a sporadic disease. Nevertheless, genetic associations involving the genes *SNCA, MAPT, PRNP*, and *COQ2* have been discussed, but without definitive evidence (Nakahara et al., 2023). Genome-wide association studies have yielded heterogeneous results, identifying potential risk loci in the gene regions *ZIC4, GAB1, lnc-LRRC49-3, TENM2*, and *RABGEF1* (Chia et al., 2024; Hopfner et al., 2022).

Differences in the conformation (“strains”) of pathological α-synuclein across synucleinopathies may influence cellular vulnerability and propagation patterns (Reddy and Dieriks, 2022). Experimental data suggest MSA-specific strain properties with enhanced seeding capacity and prion-like intercellular transmissibility. These findings might provide an explanation for the clinicopathological divergence between MSA and other synucleinopathies such as PD and DLB (Watts et al., 2013).

MSA has traditionally been defined as a primary oligodendroglial α-synucleinopathy, with GCIs representing its neuropathological hallmark. However, recent studies increasingly support the reframing of MSA as a combined neuronal–oligodendroglial α-synucleinopathy (Wiseman et al., 2025a). While GCIs remain pathognomonic, the extent of neurodegeneration appears to correlate more closely with neuronal α-synuclein pathology than with oligodendroglial cell loss. In particular, Wiseman et al. demonstrated that α-synuclein fibrils in MSA neurons can translocate from the cytoplasm into the nucleus, where they are associated with nuclear envelope disruption, lamin depletion, and neuronal cell death. In contrast, oligodendroglial inclusions, although abundant, are not accompanied by a proportional loss of oligodendroglial cell bodies. Based on these findings, the authors propose redefining MSA as a “neuronal nuclear and oligodendroglial α-synucleinopathy” (Wiseman et al., 2025a).

Ultrastructural analyses revealed distinct inclusion morphologies across oligodendrocytes and neurons, which might reflect a cell type–specific and multi-stage pathogenic process (Böing et al., 2024).

Rather than contradicting the central role of oligodendroglia, these findings support a two-compartment model of disease pathogenesis. In this framework, oligodendrocytes are critical for GCI formation, myelin dysfunction, and the establishment of a permissive microenvironment for pathological α-synuclein strains, whereas neuronal α-synuclein species may represent the more directly neurotoxic aggregates driving cell death (Kaji et al., 2020).

This concept is further supported by studies that reported increased SNCA gene and tubulin polymerization promoting protein 25α (TPPP/p25) transcripts in oligodendroglial inclusions (Kon et al., 2024). TPPP/p25 seems to stimulate α-synuclein aggregation and relocalize from myelin to GCIs in early disease stages (Lindersson et al., 2005). Moreover, TPPP/p25 can promote the formation of a distinct, more pathogenic α-synuclein strain (Song et al., 2007).

An unresolved pathomechanism concerns the directionality of α-synuclein propagation. Increasing evidence supports the hypothesis that α-synuclein is transferred from neurons to oligodendrocytes. This neuron-to-oligodendrocyte transmission model was further strengthened by recent findings demonstrating that TLR2-mediated mechanisms may facilitate the spread of α-synuclein and contribute to GCI formation in both mouse and human models (Wang et al., 2025). Thus, oligodendroglial pathology and neuronal α-synuclein toxicity might be interconnected aspects of a unified disease process.

Mitochondrial dysfunction is considered another contributing pathomechanism. Variants in the *COQ2* gene, which encodes a key enzyme in coenzyme Q10 biosynthesis, may impair the electron transport chain, reduce ATP production, and increase oxidative stress. COQ2 variants were reported as risk-associated in Japanese cohorts, while follow-up genetic analyses showed population-dependent effects and variable replication (Ronchi et al., 2016).

Chronic neuroinflammatory activation is suspected to act as an amplifying mechanism of neurodegeneration. Aggregated α-synuclein activates microglia and astrocytes, triggering the release of pro-inflammatory cytokines, complement factors, and reactive oxygen species. This inflammatory milieu may further accelerate neuronal damage. Astro- and microgliosis occur preferentially in affected brain regions and correlate with the density of glial inclusions. In addition, disturbances of iron metabolism, reduced trophic support, oligodendrocyte maturation deficits, mitochondrial dysfunction, and impaired insulin signaling pathways are discussed as possible contributing factors, which may also represent potential therapeutic targets (Vieira et al., 2015; Leńska-Mieciek et al., 2023).

## Clinical presentation

**Figure 1:**
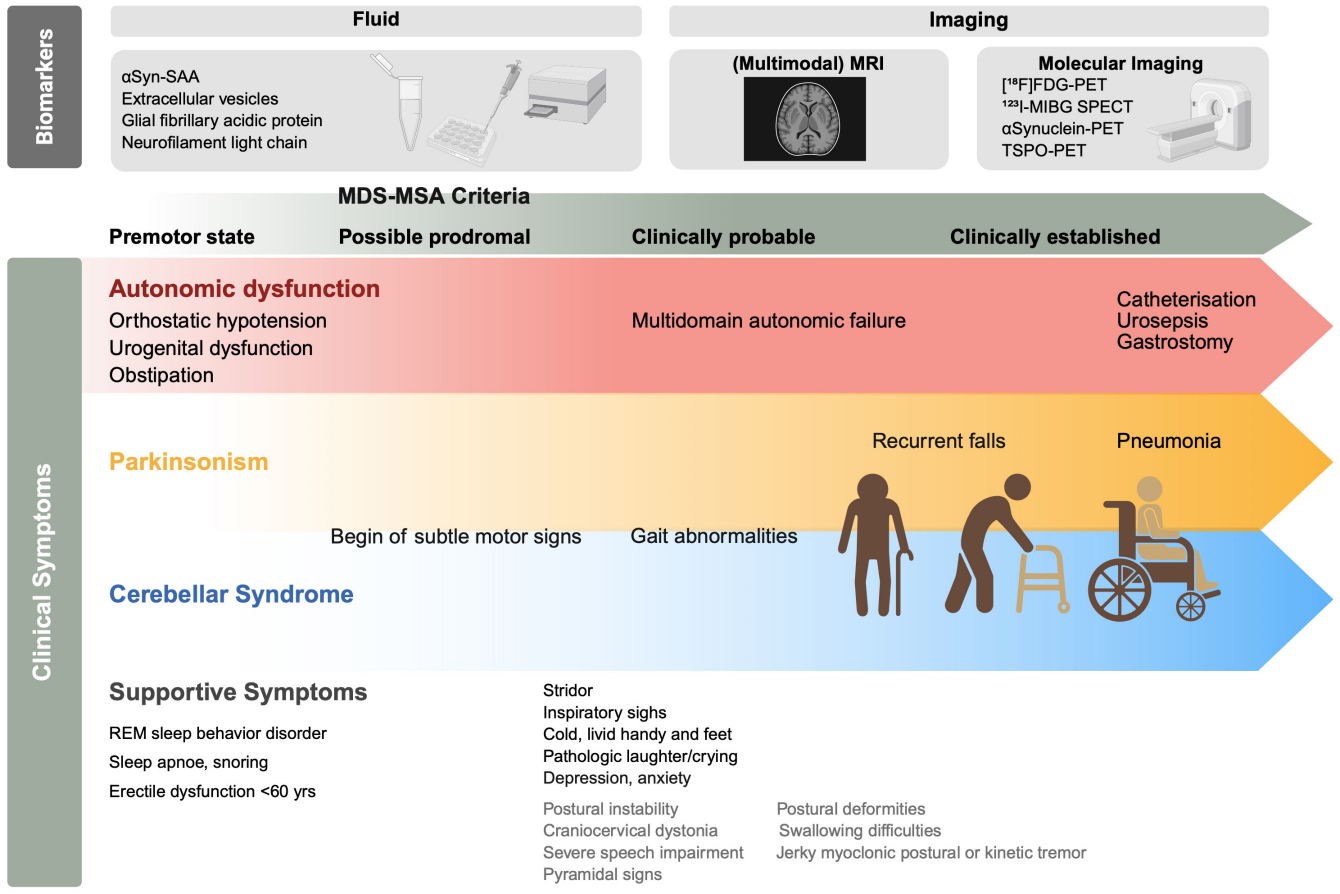
Clinical manifestations and biomarkers in MSA. MSA manifests as a variable spectrum of the core symptom complexes parkinsonism, cerebellar ataxia, and autonomic dysfunction. The clinical diagnostic criteria proposed by the Movement Disorder Society (MDS) define four levels of diagnostic certainty: neuropathologically established MSA, clinically established MSA, clinically probable MSA, and the research category of possible prodromal MSA. A premotor phase may precede these stages by several years. In addition to clinical criteria, several established and emerging biomarkers support the diagnosis of MSA. αSyn-SAA, α-synuclein seed amplification assays; [18F]FDG-PET, Fluorodeoxyglucose Positron Emission Tomography; 1^2^3I-MIBG SPECT, Iodine-123 Metaiodobenzylguanidine Single-Photon Emission Computed Tomography; MDS-MSA Criteria, clinical diagnostic criteria for MSA by the Movement Disorder Society; TSPO PET, Translocator Protein Positron Emission Tomography.

MSA manifests as a varying spectrum of parkinsonism, cerebellar ataxia, and autonomic dysfunction (Halliday et al., 2011; Wenning et al., 2022) (Figure 1). Common manifestations of this spectrum are classified as the predominantly parkinsonian subtype (MSA-P) or the cerebellar subtype (MSA-C), both accompanied by autonomic dysfunction (Fanciulli et al., 2019). However, a wide clinical spectrum exists, including motor-only phenotypes with slower disease progression (Wilkens et al., 2025). As symptoms vary in severity and onset, early and accurate diagnosis remains challenging with misdiagnosis rated of up to 35% (Koga et al., 2015).

Factors associated with a rapid disease progression include early autonomic dysfunction, REM sleep behavior disorder (RBD), stridor, and early dysphagia (Tada et al., 2007).

### Parkinsonian syndrome

The parkinsonian syndrome in MSA progresses faster than in PD, is typically symmetric, and responds poorly to levodopa (Gilman et al., 2008). Tremor is less frequent and often atypical; characteristic manifestations such as a “jerky tremor” or myoclonus may be observed. Mixed hypokinetic–ataxic–spastic dysarthria and early dysphagia are typical features (Wenning et al., 1994; Rusz et al., 2019).

### Dystonia

Dystonia is usually asymmetric, presenting in the craniocervical region or distal limbs, and may be induced or exacerbated by levodopa treatment (Boesch et al., 2002).

### Cerebellar ataxia

Cerebellar ataxia occurs in 36–64% of all MSA patients and typically presents with a broad-based gait. Additional features include limb ataxia (47–53%), scanning dysarthria (49–69%), and oculomotor abnormalities such as hypermetric saccades and gaze-evoked nystagmus (Köllensperger et al., 2010).

### Autonomic dysfunction

Autonomic dysfunction includes neurogenic orthostatic hypotension (OH), sexual and bladder dysfunction, constipation, and impaired sweating. Pure autonomic failure (PAF) may precede motor symptoms by several years (Coon et al., 2020).

OH is defined as a systolic blood pressure drop of >20 mmHg within 3 min of standing during Schellong or tilt-table test, usually accompanied by a diastolic blood pressure decrease of ≥10 mmHg (Freeman et al., 2011).

Neurogenic bladder dysfunction, such as detrusor overactivity with urgency incontinence, postvoid residual urine or increased sphincter tone, affects approximately 80% of MSA patients (Low et al., 2015). It increases the risk of recurrent urinary tract infections, hydronephrosis, and postrenal renal failure (Panicker et al., 2025). Sexual dysfunction occurs in both sexes and can be an early symptom (Köllensperger et al., 2010).

### Supportive symptoms

Supportive symptoms (Figure 1) support the MSA diagnosis, but are considered unspecific, especially in the early disease stages (Köllensperger et al., 2010).

## Prodromal MSA: redefining disease onset

Evidence supports the existence of a prodromal phase of MSA, representing a significant conceptual shift (Wenning et al., 2022). Approximately one third of patients with isolated pure autonomic failure (PAF) convert to a synucleinopathy. A normal supine plasma norepinephrine level may serve as a predictor of such conversion (Coon et al., 2020). Isolated RBD is a well-established prodromal condition of α-synucleinopathies, with reported conversion rates of up to 7.5% within 5–7 years (Poewe et al., 2022). However, while a proportion of patients with PAF convert to synucleinopathies, it does not allow for a reliable differentiation between MSA, PD, and DLB at this stage. The category of possible prodromal MSA within the clinical diagnostic criteria aims to capture this disease stage in prospective studies (Wenning et al., 2022).

## Diagnostic criteria

The clinical diagnostic criteria for MSA by the Movement Disorder Society (MDS) define four levels of diagnostic certainty: neuropathologically established MSA, clinically established MSA, clinically probable MSA, and the research category of possible prodromal MSA (Figure 1). Histopathological confirmation remains the diagnostic gold standard (Wenning et al., 2022).

A central limitation of these criteria is the trade-off between sensitivity and specificity. The diagnosis of a clinically established MSA requires clinical symptoms which manifest in advanced disease stages, resulting in high specificity but low sensitivity. Conversely, clinically probable MSA allows earlier diagnosis but at the expense of diagnostic certainty, which represents a major limitation for early therapeutic interventions. Reported specificity and sensitivity of the criteria range from 74 to 99% and 64 to 77% for clinically probable MSA, and from 99 to 100% and 16 to 22% for clinically established MSA, respectively (Wilkens et al., 2024).

## Biomarkers

Currently, no single biomarker provides sufficient diagnostic certainty for clinical diagnosis of MSA, particularly in early disease stages. Therefore, biomarkers should be interpreted in combination with the clinical symptoms, disease course, autonomic testing, and exclusion of relevant differential diagnoses. A pragmatic diagnostic approach may rely on the integration of core clinical features with supportive imaging, functional testing and fluid- or tissue-based biomarkers, rather than on isolated test results.

### Functional tests

A key feature distinguishing MSA from PD is poor levodopa responsiveness, defined by either anamnestic self-report or < 30% improvement in MDS-UPDRS III despite high-dose levodopa. Acute levodopa challenge testing is not recommended due to limited predictive value (Wenning et al., 2022).

Assessment of autonomic function is mandatory for diagnosis. OH is evaluated by active standing or tilt-table testing. Regarding urogenital dysfunction, postvoid residual volume >100 mL is the most specific marker of urogenital dysfunction. Urodynamic testing remains the diagnostic gold standard for diagnosing neurogenic urinary tract dysfunction (Panicker et al., 2025).

Additional tests to aid differential diagnosis include olfactory testing (Wenning et al., 2022), thermoregulatory testing to confirm anhidrosis (Pellecchia et al., 2020) and electromyography of the external anal sphincter to reveal denervation. Laryngoscopy shows involuntary arytenoid movements in approximately 90% of MSA patients, but rarely in PD (Calandra-Buonaura et al., 2021).

### Imaging biomarkers

The analysis of structural cranial MRI is an established biomarker, and detection of typical pathologies using 3-Tesla cranial MRI is part of the MDS diagnostic criteria. Characteristic findings include putaminal atrophy and/or T2 hypointensity, atrophy of infratentorial structures (pons, middle cerebellar peduncle, medulla oblongata, cerebellum), and the “hot cross bun” sign (Wenning et al., 2022). Although this modality shows high specificity, its sensitivity is limited, especially in early disease stages (Pavy-Le et al., 2016). Longitudinal MRI studies consistently demonstrate rapid, region-specific neurodegeneration affecting the putamen, pons, cerebellum, and cerebellar white matter, with progression rates exceeding those observed in PD (Paviour et al., 2006; Krismer et al., 2024b; Fabbri et al., 2026).

Advanced imaging techniques such as diffusion tensor imaging, iron-sensitive MRI and automated analyses demonstrate improved differentiation between MSA and PD at the group level. Diffusion-weighted MRI and diffusion tensor imaging demonstrate increased diffusivity and microstructural abnormalities in the posterior putamen and middle cerebellar peduncle and enable reliable differentiation of MSA from PD (Bajaj et al., 2017; Pasquini et al., 2022; Reginold et al., 2014). Iron-sensitive MRI techniques, such as T2^*^ imaging and quantitative susceptibility mapping, demonstrate abnormal iron deposition in the posterior putamen, globus pallidus, substantia nigra, and dentate nucleus, which are highly specific imaging markers. For example, these methods can be helpful to distinguish MSA-P from PSP, as MSA-P is characterized by predominant iron accumulation in the posterolateral putamen, whereas PSP shows more pronounced deposition in the globus pallidus (Bajaj et al., 2017; Nobileau et al., 2023). For example, the advanced diffusion techniques, such as free-water imaging, fixel-based analysis, and tractography, detect disease-specific microstructural alterations in the putamen and MCP and allow robust observer-independent differentiation (Planetta et al., 2015; Archer et al., 2019; Prodoehl et al., 2013). Automated and machine-learning analyses of structural and diffusion MRI allow observer-independent identification of MSA-specific atrophy and microstructural patterns and score excellent diagnostic performance (Vaillancourt et al., 2025; Chougar et al., 2024). Nevertheless, the translation of those imaging techniques into routine clinical practice remains limited by lack of standardization, technical complexity, and insufficient validation in early-stage disease.

#### Functional imaging techniques

Functional imaging techniques provide information beyond structural degeneration and are increasingly relevant for early diagnosis and research applications. [18F]FDG-PET reveals phenotype-dependent hypometabolism in the pons, cerebellum, or putamen and can differentiate PD from atypical Parkinson syndromes with a high diagnostic accuracy of up to 90% (Meyer et al., 2017), but it does not reliably distinguish MSA-C from ataxias. ^123^I-MIBG myocardial SPECT visualizes cardiac sympathetic innervation and aids differentiation between MSA, PD, DLB, and PAF. Uptake is usually preserved in MSA, reflecting a central preganglionic lesion (Eckhardt et al., 2022).

### Molecular imaging

Molecular imaging adds to the diagnostic and pathophysiological assessment of MSA by visualizing functional and cellular changes. PET-based glial imaging proves to be a tool to assess astrogliosis and microgliosis as markers of neuroinflammation. Initial studies that utilized monoamine oxidase-B (MAO-B) tracers indicated region-specific astroglial activation in MSA (Horimoto et al., 2022; Ballweg et al., 2023). However, interpretation requires caution, as some tracers rely on off-target binding or need further validation. More robust evidence is available for PET ligands targeting the translocator protein (TSPO), a marker of microglial and astroglial activation. A multicenter study using [11C]PBR28 demonstrated markedly increased TSPO binding in MSA compared with PD, particularly in the putamen and cerebellar white matter, suggesting neuroinflammation as a key disease feature (Jucaite et al., 2022).

A promising step toward direct *in vivo* assessment of MSA pathology may be the use of PET ligands targeting α-synuclein. Tracers such as [18F]SPAL-T-06, [18F]ACI-12589, and [18F]C05-05 demonstrated increased uptake in disease-relevant regions in early studies, although comprehensive clinical validation remains pending (Matsuoka et al., 2022; Smith et al., 2023; Endo et al., 2024).

### Fluid biomarkers

The development of reliable fluid biomarkers for clinical routine is essential not only for integrating MSA into a biologically grounded classification of synucleinopathies (Höglinger et al., 2024), but also for enabling early diagnosis and the objective assessment of disease-modifying interventions (Krismer et al., 2024a).

Among currently available markers, neurofilament light chain (NfL) and glial fibrillary acidic protein (GFAP) have been most consistently studied. Both reflect key pathological processes—axonal degeneration and astroglial activation—and are typically elevated in MSA compared with PD (Katzdobler et al., 2024). However, their diagnostic utility is limited by insufficient specificity, as similar changes can be observed across a range of neurodegenerative conditions (Katzdobler et al., 2024).

Current research focuses on α-synuclein seed amplification assays (αSyn-SAA), which allow ultrasensitive detection of misfolded α-synuclein in cerebrospinal fluid, blood, and skin (Siderowf et al., 2023; Poggiolini et al., 2022). These assays detect α-synuclein pathology in PD and DLB. Modified assay protocols demonstrated partial discrimination between PD and MSA (Reddy and Dieriks, 2022; Shahnawaz et al., 2020). According to different assays, αSyn-SAA are negative or show a much smaller increase in MSA, reflecting structural and biophysical differences of MSA-specific α-synuclein strains (Wiseman et al., 2025b; Luan et al., 2022). Hence, a key limitation has become evident: biomarker platforms optimized for PD are not directly transferable to MSA.

Beyond protein-based markers, approaches to analyze metabolomic profiles of cerebrospinal fluid and plasma identified alterations which reflect mitochondrial dysfunction and impaired energy metabolism. Eventhough these approaches are still in an exploratory phase and lack standardization, they might serve as potential complementary biomarker modalities (Kaji et al., 2020).

A novel diagnostic strategy involves the analysis of extracellular vesicles, particularly exosomes, as carriers of disease-relevant proteins. Exosomes are released by multiple cell types, including neurons and oligodendrocytes, and can be detected in peripheral biofluids such as plasma, cerebrospinal fluid, and saliva (Doyle and Wang, 2019). They are capable of transporting α-synuclein, which suggests that cell type–specific exosomal cargo may reflect ongoing pathological processes in the central nervous system (Donadio et al., 2026a). Studies investigating α-synuclein levels in neuron- and oligodendrocyte-derived exosomes have yielded inconsistent results. Current findings are inconsistent: some studies report reduced α-synuclein levels in oligodendroglia-derived exosomes in MSA, with unchanged levels in neuron-derived vesicles, whereas others describe increased α-synuclein in both neuronal and oligodendroglial exosomes. These discrepancies likely reflect methodological differences in exosome isolation and characterization, as well as biological heterogeneity (Kaji et al., 2020).

### Skin biomarkers

The detection of phosphorylated α-synuclein in skin samples using immunofluorescence techniques has turned out to be another promising biomarker (Doppler et al., 2018; Donadio et al., 2023, 2026b). Several studies report a high specifity for PD of 100% and a sensitivity of up to 81%, even in early disease stages where clinical diagnostic criteria are not met yet. The cell-type specific distribution patterns of phosphorylated α-synuclein have been investigated in different synucleinopathies (Donadio et al., 2020). Surprisingly, fibrillar phosphorylated α-synuclein in Schwann cells has found to be a pathological hallmark MSA. In other synucleinopathies (PD/DLB) phosphorylated α-synuclein has only been found in neurons, mirroring the distribution of α-synuclein in the brain (Donadio et al., 2023).

In summary, structural MRI and autonomic testing currently represent the only clinically established supportive biomarkers for MSA diagnosis (Wenning et al., 2022). Functional imaging, NfL/GFAP, α-synuclein seed amplification assays, exosomal markers, and skin biopsy may increase diagnostic confidence in selected cases, but their role in routine diagnosis remains limited due to incomplete validation, variable availability, and uncertain specificity in early disease. Therefore, a multimodal diagnostic strategy in which clinical examination remains the foundation, supported by targeted biomarker testing according to the dominant phenotype and differential diagnostic question, should be the main approach.

### Disease monitoring

To evaluate disease-modifying therapies, sensitive clinical scales are required that reliably capture functionally relevant changes. The Unified Multiple System Atrophy Rating Scale is currently the most used outcome measure in clinical trials. A recent revision seeks to improve its sensitivity and patient-centeredness (Wenning et al., 2004). Automated digital biomarkers—such as data derived from wearable sensors—may complement clinical endpoints and allow for objective, continuous monitoring of disease progression (Warmerdam et al., 2020).

## Therapy

The management of MSA remains largely symptomatic and multidisciplinary. Pharmacological treatment of parkinsonism is often limited by poor and transient response to levodopa (Low et al., 2015), while therapies for autonomic dysfunction, sleep disorders, and urogenital symptoms provide only partial relief. Care should be multidisciplinary, involving physiotherapy, occupational therapy, speech therapy, and nursing staff, to preserve quality of life. Owing to the rapid disease progression, early palliative co-management should be considered (Oliver et al., 2024; Jensen et al., 2022).

### Disease modifying therapies

Because no validated predictive biomarkers or modifiable risk factors are currently available, disease-modifying strategies for MSA focus on early intervention to target core pathogenic mechanisms, particularly α-synuclein aggregation and spread.

α-Synuclein–directed therapies include active and passive immunization. The vaccine PD01A was safe and immunogenic in a phase-1 study, reducing oligomeric α-synuclein in CSF (NCT02270489). The synthetic vaccine UB-312 (NCT05634876) and the monoclonal antibody amlenetug (Lu AF82422) are currently under investigation, the latter in the phase-3 MASCOT trial (NCT06706622) (Jucaite et al., 2022).

Small molecules with anti-aggregative or antioxidant properties, such as emrusolmin (TEV-56286), inhibit oligomer formation and are being evaluated in a phase-2 trial (NCT06568237). Antisense oligonucleotides targeting α-synuclein synthesis (e.g., ION-464) are also under study (Stamler et al., 2024).

Given the role of neuroinflammation, anti-inflammatory strategies are being explored. The myeloperoxidase inhibitor verdiperstat (BHV-3241) failed to meet its primary endpoint in the phase-3 M-STAR trial (NCT03952806), while combination approaches (e.g., anti-α-synuclein antibody CD5-D5 plus lenalidomide) showed benefit in experimental models (Mészáros et al., 2020).

Because neurodegeneration is thought to occur secondary to oligodendroglial dysfunction, neuroprotective approaches remain central. Mesenchymal stem cells exhibit anti-inflammatory and neuroprotective effects, with delivery strategies under investigation (NCT05698017, NCT01716481). mTOR inhibition with sirolimus may enhance autophagy and α-synuclein clearance (Mészáros et al., 2020). Antioxidants and gene-therapy approaches (NRF2, GDH2, EAAT2) demonstrated preclinical efficacy (NCT03403309, NCT07081841). Ubiquinol supplementation, prompted by COQ2 mutations, slowed disease progression in a phase-2 study (Ronchi et al., 2016). New targets, such as Abelson tyrosine kinase inhibitors and GLP-1 receptor agonists, may provide additional neuroprotective effects (NCT05424276). Early, causally oriented intervention is considered essential to limit secondary neurodegeneration.

## Synopsis

**Figure 2:**
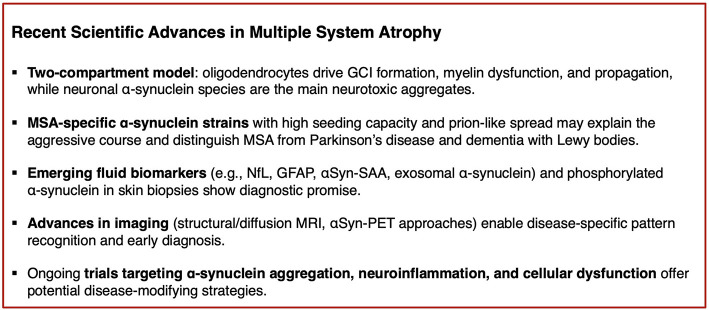
Recent advances in multiple system atrophy.

MSA is a rapidly progressive synucleinopathy characterized by autonomic failure, parkinsonism, and cerebellar ataxia, with oligodendroglial α-synuclein inclusions as its pathological hallmark. Recent advances challenge the traditional glio-centric view and support a combined neuronal–oligodendroglial disease model in which neuronal α-synuclein toxicity plays a central role in neurodegeneration. Strain-specific α-synuclein biology, neuron-to-oligodendrocyte propagation, mitochondrial dysfunction, and neuroinflammatory processes are discussed as pathogenetic mechanisms (Böing et al., 2024; Kaji et al., 2020; Kon et al., 2024; Song et al., 2007; Wang et al., 2025) (Figure 2).

The clinical diagnosis of MSA remains difficult due to varying clinical symptoms and the lack of validated early biomarkers. The clinical examination remains the cornerstone of diagnosis, while biomarkers should be used to increase diagnostic confidence, support differential diagnosis, and identify patients at earlier disease stages. At present, structural MRI and autonomic testing have the strongest role in routine clinical practice, whereas advanced imaging, fluid biomarkers, α-synuclein seed amplification assays, and skin biopsy remain promising but incompletely validated tools (Reddy and Dieriks, 2022; Wang et al., 2025; Shahnawaz et al., 2020; Stamler et al., 2024). A multimodal approach to combine the clinical symptoms, disease progression, autonomic assessment, MRI findings, and selected molecular or tissue biomarkers is therefore the most pragmatic strategy for improving diagnostic accuracy.

Emerging disease-modifying strategies targeting α-synuclein, neuroinflammation, and cellular dysfunction highlight the need for early, biologically defined diagnosis to improve therapeutic outcomes as key priorities for improving outcomes in MSA, especially in prodromal and early-stage MSA, where therapeutic intervention is most likely to be effective.
